# The discrete adjoint method for parameter identification in multibody system dynamics

**DOI:** 10.1007/s11044-017-9600-9

**Published:** 2017-11-03

**Authors:** Thomas Lauß, Stefan Oberpeilsteiner, Wolfgang Steiner, Karin Nachbagauer

**Affiliations:** Faculty of Engineering and Environmental Sciences, University of Applied Sciences Upper Austria, Stelzhamerstrasse 23, 4600 Wels, Austria

**Keywords:** Adjoint method, Discrete adjoint method, Parameter identification

## Abstract

The *adjoint method* is an elegant approach for the computation of the gradient of a cost function to identify a set of parameters. An additional set of differential equations has to be solved to compute the adjoint variables, which are further used for the gradient computation. However, the accuracy of the numerical solution of the adjoint differential equation has a great impact on the gradient. Hence, an alternative approach is the *discrete adjoint method*, where the adjoint differential equations are replaced by algebraic equations. Therefore, a finite difference scheme is constructed for the adjoint system directly from the numerical time integration method. The method provides the exact gradient of the discretized cost function subjected to the discretized equations of motion.

## Introduction

In the last few years the complexity of the multibody systems has grown tremendously. In particular, industrial simulations of large systems include a high number of bodies, resulting in a vast number of degrees of freedom. In general, the bodies are linked to the ground or to other bodies by formulating algebraic constraint equations.

In most cases, multibody systems are described in descriptor form, given by a system of differential algebraic equations (DAE)

1 $$ \begin{aligned} \mathbf{M}(\mathbf{q},\mathbf{u}) \ddot{ \mathbf{q}} + \mathbf{C}_{\mathbf{q}}^{\mathsf{T}}(\mathbf{q})\boldsymbol { \lambda}&= \mathbf{Q}(\mathbf{q},\dot{\mathbf{q}},\mathbf{u},t), \\ \mathbf{C}(\mathbf{q},t) &= \mathbf{0}, \end{aligned} $$

in which $\mathbf{q}$ denotes the generalized coordinates, and $\dot {\mathbf{q}}$ and $\ddot{\mathbf{q}}$ its time derivatives, $\mathbf {M}$ is the symmetric system mass matrix, and $\mathbf{Q}$ represents the vector of generalized and gyroscopic forces. Due to the algebraic constraints $\mathbf{C}(\mathbf{q},t) = \mathbf{0}$, the equations of motion have to be extended by constraint forces of the form $\mathbf{C}_{\mathbf{q}}^{\mathsf{T}}\boldsymbol{\lambda }$, where $\mathbf{C}_{\mathbf{q}}$ represents the constraint Jacobian matrix, and the vector $\boldsymbol{\lambda}$ includes the Lagrange multipliers. Moreover, $\mathbf{u}$ is a vector of parameters influencing the system behavior. We consider an optimization problem for the multibody system, which can be described in the general form as follows: Find the vector of unknown parameters $\mathbf{u}$ such that the cost function given by

2 $$ J ( \mathbf{u} ) = \int_{0}^{t_{f}}\frac{1}{2} \bigl( \textbf {s} ( \mathbf{q},\dot{\mathbf{q}},\ddot{\mathbf{q}},\boldsymbol {\lambda} ) -\bar{ \textbf{s}} ( t ) \bigr) ^{2} \,\mathrm{d}t $$

is minimized, where $t_{f}$ is the final end time. The integrand represents the mean deviation between a system output $\textbf {s}(\mathbf{q},\dot{\mathbf{q}},\ddot{\mathbf{q}},\boldsymbol{\lambda })$ and a given measured signal $\bar{\textbf{s}}(t)$.

Various authors formulate the parameter identification problem as an optimization task. Vyasarayani et al. [21] solve the underlying optimization problem using a combination of the Gauss–Newton and single shooting methods. Therein, homotopy continuation is used to find a global minimum of the cost function. The gradient is computed by the sensitivities, and the Hessian is only of first-order accuracy since second-order terms are neglected.

Serban et al. [19] also realized the parameter identification in multibody systems by minimizing a cost function by the Levenberg–Marquardt method. The derivatives that are required for the optimization are computed through sensitivity analysis. In addition, a local identifiability test is developed in this contribution.

Oberpeilsteiner et al. [15] designed an optimal input by maximizing the information content of the parameters to identify. The required Jacobian matrix are computed with the adjoint method, and the optimization is done with the gradient method. Finally, the optimal input is used for a parameter identification.

Apart from the previous described methods, the adjoint method is already used in a wide range of parameter identification problems in engineering sciences. Especially, in the field of multibody systems, the computation of the gradient of the cost function, as, for example, in (2), is often the bottleneck for computational efficiency, and the adjoint method serves as the most efficient strategy in this case. The basic idea of the adjoint method is the introduction of additional *adjoint* variables determined by a set of adjoint differential equations from which the gradient can be computed straightforwardly. This main idea directly corresponds to the gradient technique for trajectory optimization pioneered by Bryson and Ho [3]. There are two strategies for this purpose: the equations of motion of the multibody system and adjoint equations may either be separately discretized from their representations as differential-algebraic equations, or, alternatively, the equations of motion of the multibody system may be discretized first, whereas the discrete adjoint equations are derived directly from the discrete multibody system equations; for more details, see [3].

The piecewise adjoint method presented in [18] formulates the coordinate partitioning underlying ordinary differential equations as a boundary value problem, which is solved by multiple shooting methods. The sensitivity analysis for differential-algebraic and partial differential equations using adjoint methods has also been in the focus of the group around Petzold, Cao, Li, and Serban [16]. The adjoint method has been used for sensitivity analysis in multibody systems as well by Eberhard [4], presenting a continuous, hybrid form of automatic differentiation. In [20], the use of the adjoint method for solving dynamical inverse problems is described, but rather academic examples are discussed. A recent paper [11] shows how the adjoint method can be applied efficiently to a multibody system described by differential-algebraic equations of index three. It also presents the structure of the adjoint equations depending on the Jacobian matrices of the system equations. However, the numerical solution of the adjoint system presented in [11] raises several questions concerning stability and accuracy with respect to time discretization.

An alternative and more natural approach is the discrete adjoint method (DAM), which constructs a finite difference scheme for the adjoint system directly from the numerical procedure used to solve the equations of motion. The method delivers the exact gradient of the discretized cost function subjected to the discretized equations of motion. Instead of using automatic differentiation techniques [1], the Jacobian matrices can be determined analytically for multibody systems in a very simple structure if redundant generalized coordinates are used to describe the motion of the bodies. In [7] the discrete adjoint method is derived and applied for optimal control problems only, focusing especially on the description of the adjoint method for explicit and implicit solvers for optimal control applications, as well as the interpolation of the gradients and the Hessian (BFGS).

The current paper focuses on parameter identification of multibody systems. The discrete adjoint equations are derived for the computation of the gradient of the cost function using the HHT-solver [6, 13] for the solution of the system equations.

The advantage of the presented method is that the cost function may also depend on the accelerations if the discrete adjoint method is used. The reason is that the accelerations are included in the state vector of the HHT-solver. In contrast to the discrete adjoint method, in the continuous approach, the accelerations have to be expressed by the equations of motion, leading to a complex Jacobian matrix [12]. Practically speaking, the new approach allows us to use measured data from acceleration sensors in a straightforward manner as a reference trajectory in the cost function for the parameter identification.

## Discrete adjoint method for implicit time integration methods

The discrete adjoint method is an elegant and efficient way to compute the gradient of a cost function. Following [7], the discretized multibody system equations can be rewritten in the form

3 $$ \mathbf{f}(\mathbf{x}_{i+1},\mathbf{x}_{i},t_{i+1},t_{i}, \mathbf{u})= \mathbf{0}, \quad\mathbf{x}_{0} \text{ given}, \quad i = 0,\dots,N-1, $$

in which $\mathbf{u}\in\mathbb{R}^{m}$ denotes the vector of parameters to identify. The sequence of state vectors $\mathbf{x}_{0}, \dots, \mathbf{x}_{N} \in\mathbb{R}^{n}$ are given at times $t_{0}, \dots, t_{N} \in\mathbb{R}$. A discretized version of the cost function in (2) is given by

4 $$ J(\mathbf{u}) = \frac{1}{2}\sum_{i=0}^{N-1} \eta_{i} \bigl( \textbf {s} ( \mathbf{x}_{i} ) -\bar{ \textbf{s}} ( t_{i} ) \bigr) ^{2}, $$

where $\eta_{i}$ is a weighting factor. Hence, the goal is to find a set of parameters $\mathbf{u}$ such that the scalar cost function (4) is minimized. To introduce the adjoint gradient computation, the cost function $J$ is extended by zero terms, representing the system equation (3), and reads

5 $$ \bar{J} = \sum_{i=0}^{N-1} \biggl\{ \frac{1}{2}\eta_{i} \bigl( \textbf {s} ( \mathbf{x}_{i} ) -\bar{\textbf{s}} ( t_{i} ) \bigr) ^{2} + \mathbf{p}_{i+1}^{\mathsf{T}}\mathbf{f}(\mathbf {x}_{i+1}, \mathbf{x}_{i}, t_{i+1}, t_{i}, \mathbf{u}) \biggr\} , $$

where $\mathbf{p}_{1}, \dots, \mathbf{p}_{N}$ denote the *adjoint variables*. Due to (3), $\bar{J}$ and $J$ are equal for any choice of the adjoints, and so the corresponding gradients with respect to the parameters to identify are also equal. We will further choose $\mathbf{p}_{i}$ such that the gradient computation becomes as easy as possible. Hence, the variation of the cost function is given by

6 $$ \begin{aligned} \delta\bar{J} &= \sum _{i=0}^{N-1} \biggl\{ \eta_{i} \bigl[ \textbf {s}_{i}-\bar{\textbf{s}} ( t_{i} ) \bigr] ^{\mathsf{T}} \frac {\partial\textbf{s}_{i}}{\partial\mathbf{x}_{i}}\delta\mathbf {x}_{i} \\ &\quad{}+ \mathbf{p}_{i+1}^{\mathsf{T}} \biggl( \frac{\partial\mathbf {f}_{i+1}}{\partial\mathbf{x}_{i+1}} \delta\mathbf{x}_{i+1} + \frac {\partial\mathbf{f}_{i+1}}{\partial\mathbf{x}_{i}} \delta\mathbf {x}_{i} + \frac{\partial\mathbf{f}_{i+1}}{\partial\mathbf{u}} \delta \mathbf{u} \biggr) \biggr\} \end{aligned} $$

with the abbreviations $\mathbf{f}_{i+1} = \mathbf{f}(\mathbf{x}_{i+1}, \mathbf{x}_{i}, t_{i+1}, t_{i}, \mathbf{u})$ and $\textbf{s}_{i} = \textbf{s}(\mathbf{x}_{i})$ and the corresponding Jacobian matrix $\partial\textbf{s}_{i}/\partial\mathbf {x}_{i}$, which is the partial derivative of the system output with respect to the states $\mathbf{x}$ evaluated at $\mathbf{x}_{i}$. Following the trivial index shift

7 $$ \sum_{i=0}^{N-1}\mathbf{p}_{i+1}^{\mathsf{T}} \frac{\partial\mathbf {f}_{i+1}}{\partial\mathbf{x}_{i+1}} \delta\mathbf{x}_{i+1} = \sum _{i=1}^{N}\mathbf{p}_{i}^{\mathsf{T}} \frac{\partial\mathbf {f}_{i}}{\partial\mathbf{x}_{i}} \delta\mathbf{x}_{i}, $$

the variation of the cost function $\bar{J}$ can be reformulated in terms of $\delta\mathbf{x}_{i}$ and $\delta\mathbf{u}$ as

8 $$ \begin{aligned}[b] \delta\bar{J} &= \biggl( \mathbf{p}_{1}^{\mathsf{T}} \frac{\partial\mathbf{f}_{1}}{\partial \mathbf{x}_{0}} + \eta_{0} \bigl[ \textbf{s}_{0}-\bar{ \textbf{s}} ( t_{0} ) \bigr] ^{\mathsf{T}}\frac{\partial\textbf {s}_{0}}{\partial\mathbf{x}_{0}} \biggr) \delta\mathbf{x}_{0} + \mathbf{p}_{1}^{\mathsf{T}} \frac{\partial\mathbf{f}_{1}}{\partial \mathbf{u}} \delta\mathbf{u} \\ &\quad{}+ \sum_{i=1}^{N-1} \biggl[ \biggl( \eta_{i} \bigl[ \textbf{s}_{i}-\bar{\textbf{s}} ( t_{i} ) \bigr] ^{\mathsf{T}}\frac{\partial\textbf{s}_{i}}{\partial\mathbf {x}_{i}} + \mathbf{p}_{i}^{\mathsf{T}}\frac{\partial\mathbf{f}_{i}}{\partial \mathbf{x}_{i}} + \mathbf{p}_{i+1}^{\mathsf{T}} \frac{\partial\mathbf{f}_{i+1}}{\partial \mathbf{x}_{i}} \biggr) \delta\mathbf{x}_{i} \\ &\quad{}+ \mathbf{p}_{i+1}^{\mathsf{T}}\frac{\partial\mathbf{f}_{i+1}}{\partial \mathbf{u}}\delta \mathbf{u} \biggr] + \mathbf{p}_{N}^{\mathsf{T}}\frac{\partial\mathbf{f}_{N}}{\partial \mathbf{x}_{N}} \delta\mathbf{x}_{N}. \end{aligned} $$

The adjoint state variables should be defined in such a way that the expressions in the brackets corresponding to the variation of the states vanish, and hence the complicated relations between $\delta \mathbf{x}_{i}$ and $\delta\mathbf{u}$ need not be computed. Note that $\delta\mathbf{x}_{0}$ vanishes. If we define the adjoint variables $\mathbf{p}_{i}$ by the equations

9 $$ \begin{aligned} \biggl( \frac{\partial\mathbf{f}_{N}}{\partial\mathbf{x}_{N}} \biggr) ^{\mathsf{T}}\mathbf{p}_{N} &= \mathbf{0}, \\ \biggl( \frac{\partial\mathbf{f}_{i}}{\partial\mathbf{x}_{i}} \biggr) ^{\mathsf{T}}\mathbf{p}_{i} &= - \eta_{i} \biggl( \frac{\partial\textbf {s}_{i}}{\partial\mathbf{x}_{i}} \biggr) ^{\mathsf{T}} \bigl[ \textbf {s}_{i}-\bar{\textbf{s}} ( t_{i} ) \bigr] - \biggl( \frac {\partial\mathbf{f}_{i+1}}{\partial\mathbf{x}_{i}} \biggr) ^{\mathsf {T}}\mathbf{p}_{i+1}, \end{aligned} $$

which can be solved successively for $\mathbf{p}_{i}$, starting with the boundary condition for $\mathbf{p}_{N}$ and proceeding with $i=(N-1),\dots,1$, then the variation of the cost function is reduced to

10 $$ \delta\bar{J} = \sum_{i=0}^{N-1} \biggl( \mathbf{p}_{i+1}^{\mathsf{T}}\frac{\partial\mathbf{f}_{i+1}}{\partial \mathbf{u}} \biggr) \delta \mathbf{u}= \biggl( \frac{\partial J}{\partial\mathbf {u}} \biggr) ^{\mathsf{T}}\delta\mathbf{u}. $$

Hence, the gradient is given by

11 $$ \frac{\partial J}{\partial\mathbf{u}} = \sum_{i=0}^{N-1} \biggl( \frac{\partial\mathbf{f}_{i+1}}{\partial\mathbf{u}} \biggr) ^{\mathsf{T}}\mathbf{p}_{i+1}. $$

In summary, to find the gradient of the cost function, we can proceed as follows. First, the difference equations (3) have to be solved forward in time with an initial guess for the actual values of the parameters $\mathbf {u}$. Furthermore, the adjoint equations (9) have to be solved backward in time starting from the boundary conditions for $\mathbf{p}_{N}$. In combination with the adjoint variables, the gradient of the cost function with respect to the parameters $\mathbf {u}$ can be evaluated from (11).

The gradient information can be used in various ways to find a set of parameters that minimizes the cost function. In the simplest form, the parameters are updated by walking in the direction of the negative gradient $-\partial J/\partial\mathbf{u}$, that is, by setting $\mathbf{u}^{\text{new}} = \mathbf{u}- \kappa ( \partial J/\partial\mathbf{u} ) $. If the number $\kappa> 0$ is sufficiently small, then the updated set of parameters always reduces the cost function $J$ (steepest-descent method).

It should be noted that the convergence of a quasi-Newton method is much better than the convergence of the steepest-descent method, especially near the optimum. Hence, it is recommended to use a quasi-Newton method where the Hessian is approximated from the gradient information, for example, with the BFGS formula. The Hessian could also be estimated from the first-order sensitivity matrix [19], but as the adjoint method circumvents its computation, this approach seems not appropriate in this context.

## Application to the HHT solver

In this section the implicit iteration scheme (3) is specified for the HHT-solver, which is a widely used time integration method in multibody system dynamics. The Jacobian matrices required for the computation of the discrete adjoint variables, and further for the gradient computation, are derived in this section.

A precursor of the HHT-method is the Newmark method [14], which approximates the positions and velocities of a second-order system by the formulas

12 $$ \begin{aligned} \mathbf{q}_{i} &= \mathbf{q}_{i-1} + h\dot{\mathbf{q}}_{i-1} + \frac {h^{2}}{2} \bigl[(1-2\beta) \ddot{\mathbf{q}}_{i-1} + 2\beta\ddot{{\mathbf {q}}}_{i}\bigr], \\ \dot{\mathbf{q}}_{i} &= \dot{\mathbf{q}}_{i-1} + h\bigl[(1- \gamma)\ddot {\mathbf{q}}_{i-1} + \gamma\ddot{{\mathbf{q}}}_{i} \bigr], \end{aligned} $$

with parameters $\beta$ and $\gamma$. Herein, $\mathbf{q}_{i}$, $\dot {\mathbf{q}}_{i}$, and $\ddot{\mathbf{q}}_{i}$ denote positions, velocities, and accelerations at $t=t_{i}$. The formulas (12) are used to discretize the motion equations (1) using the integration step size $h=t_{i}-t_{i-1}$. Choosing $\gamma=\frac{1}{2}$ and $\beta=\frac {1}{4}$, the method is equivalent to the trapezoidal rule, which is implicit and A-stable and leads to a second-order integration formula. The disadvantage of the trapezoidal rule is that no numerical damping is introduced, and so this method is impractical for stiff problems. Hence, the HHT-method was developed as an improvement, which includes numerical damping and preserves its A-stability while achieving second-order accuracy.

The method considers the second-order term in the motion equations (1) at $t=t_{i}$ whereas all other terms are weighted between $t_{i-1}$ and $t_{i}$ by using the weighting factor $\alpha$, resulting in

13 $$ \frac{1}{1+\alpha} (\mathbf{M}\ddot{\mathbf{q}})_{i} + \bigl(\mathbf {C}_{\mathbf{q}}^{\mathsf{T}}\boldsymbol{\lambda }- \mathbf{Q} \bigr)_{i} -\frac{\alpha}{1+\alpha}\bigl(\mathbf{C}_{\mathbf{q}}^{\mathsf{T}} \boldsymbol{\lambda}- \mathbf {Q}\bigr)_{i-1} = 0. $$

As indicated in [13], the parameter $\alpha$ has to be chosen in the interval $\alpha\in[-\frac{1}{3},0]$ to possess the advertised stability. The parameter $\beta$ and $\gamma$ of (12) are expressed by $\alpha$ by setting $\beta= (1-\alpha)^{2}/4$ and $\gamma= (1-2\alpha)/2$.

Finally, for constrained systems, the constraint equation satisfied at $t=t_{i}$ is

14 $$ \mathbf{C}(\mathbf{q}_{i},t_{i})= 0. $$

By introducing the state vector $\mathbf{x}_{i}^{\mathsf{T}}= [\mathbf {q}_{i}^{\mathsf{T}}\ \dot{\mathbf{q}}_{i}^{\mathsf{T}}\ \ddot {\mathbf{q}}_{i}^{\mathsf{T}}\ \boldsymbol{\lambda}_{i}^{\mathsf {T}}]=[\mathbf{q}_{i}^{\mathsf{T}}\ \mathbf{v}_{i}^{\mathsf {T}}\ \mathbf{a}_{i}^{\mathsf{T}}\ \boldsymbol{\lambda}_{i}^{\mathsf{T}}]$, Eqs. (12), (13), and (14) have the form (3) and may be solved for $\mathbf{x}_{i}$ if $\mathbf{x}_{i-1}$ is known. Rewriting (12), (13), and (14) yields

15 $$ \begin{aligned} \mathbf{f}_{1,i} &= - \mathbf{q}_{i-1} +\mathbf{q}_{i} - h\mathbf {v}_{i-1} - \frac{h^{2}}{2}\bigl[(1-2\beta) \mathbf{a}_{i-1} + 2\beta \mathbf{a}_{i}\bigr] = 0, \\ \mathbf{f}_{2,i} &= -\mathbf{v}_{i-1} + \mathbf{v}_{i} - h\bigl[(1-\gamma )\mathbf{a}_{i-1} + \gamma\mathbf{a}_{i} \bigr] = 0, \\ \mathbf{f}_{3,i} &= \frac{1}{1+\alpha} (\mathbf{M} \mathbf{a})_{i} + \bigl(\mathbf{C}_{\mathbf{q}}^{\mathsf{T}} \boldsymbol{\lambda}- \mathbf {Q}\bigr)_{i} - \frac{\alpha}{1+\alpha}\bigl( \mathbf{C}_{\mathbf{q}}^{\mathsf{T}}\boldsymbol{\lambda}- \mathbf{Q} \bigr)_{i-1} = 0, \\ \mathbf{f}_{4,i} &= \frac{1}{\beta h^{2}} \mathbf{C}(\mathbf{q}_{i}) = 0, \end{aligned} $$

where the equations are summarized in $\mathbf{f}_{i}^{\mathsf {T}}=[\mathbf{f}_{1,i}^{\mathsf{T}}\ \mathbf{f}_{2,i}^{\mathsf {T}}\ \mathbf{f}_{3,i}^{\mathsf{T}}\ \mathbf{f}_{4,i}^{\mathsf {T}}]$, which equals the right-hand side of the implicit recursive scheme (3). The Jacobian matrices $\frac{\partial\mathbf {f}_{i}}{\partial\mathbf{x}_{i}}$ and $\frac{\partial\mathbf {f}_{i+1}}{\partial\mathbf{x}_{i}}$, which are required for the computation of the adjoint variables according to (9), are given further. Note that $\mathbf{q}_{i+1} = \mathbf{q}_{i+1}(\mathbf{q}_{i}, \mathbf{v}_{i}, \mathbf{a}_{i}, \mathbf {a}_{i+1})$ due to the Newmark integration formulas (12). First, the partial derivative of the implicit iteration scheme $\mathbf{f}_{i}$ with respect to the actual states $\mathbf{x}_{i}$ at time $t_{i}$ is calculated and given by

16 $$ \frac{\partial\mathbf{f}_{i}}{\partial\mathbf{x}_{i}} = \left. \begin{pmatrix} \mathbf{I} & \mathbf{0} & -h^{2}\beta\mathbf{I} & \mathbf {0} \\ \mathbf{0} & \mathbf{I} & -h \gamma\mathbf{I} & \mathbf{0} \\ \mathbf{0} & \mathbf{0} & \frac{1}{1+\alpha} \mathbf{M}+ ( \frac{1}{1+\alpha}\mathbf{J_{M}} + \mathbf{J_{C}} - \mathbf {J_{Q}} ) \beta h^{2} - \mathbf{J_{V}}h \gamma\quad& \mathbf {C}_{\mathbf{q}}^{\mathsf{T}}\\ \mathbf{0} & \mathbf{0} & \mathbf{C}_{\mathbf{q}}& \mathbf{0} \end{pmatrix} \right \vert _{t=t_{i}} $$

with abbreviations

$$\begin{aligned} \textstyle\begin{array}{rcl@{\qquad}l} \mathbf{J_{M}}(t_{i}) &=& \frac{\partial}{\partial\mathbf{q}} ( \mathbf{M}\ddot{\mathbf{q}}) \bigg\vert _{t=t_{i}}, &\mathbf{J_{C}}(t_{i}) = \frac{\partial}{\partial\mathbf {q}} \bigl( \mathbf{C}_{\mathbf{q}}^{\mathsf {T}} \boldsymbol{\lambda} \bigr) \bigg\vert _{t=t_{i}}, \\ \mathbf{J_{Q}}(t_{i}) &= & \frac{\partial\mathbf{Q}}{\partial \mathbf{q}} \bigg\vert _{t=t_{i}}, & \mathbf{J_{V}}(t_{i}) = \frac{\partial\mathbf{Q}}{\partial\dot {\mathbf{q}}} \bigg\vert _{t=t_{i}}. \end{array}\displaystyle \end{aligned}$$

It is worth noting here that the lower right block of Jacobian matrix (16) corresponds to the Jacobian matrix (12) in Negrut et al. [13]. In this paper the constraints are scaled by a factor of $\frac{1}{\beta h^{2}}$ to improve the condition number of the Jacobian matrix. In [5] a formal proof is given, which discusses the nonsingular character of the block Jacobian matrix (16). Furthermore, for the computation of the discrete adjoint variables, the inverse of the transposed matrix (16) is required. Due to the special structure of the Jacobian matrix, the adjoint variables based on the generalized coordinates and on the generalized velocities can be calculated directly by separating the matrix. The discrete adjoint variables based on the generalized acceleration and on the Lagrange multiplier can be calculated by inverting only the right lower part of the matrix (16). Hence, only the condition number of this submatrix is of interest.

In addition, the partial derivatives of the implicit iteration scheme (3) with respect to the states $\mathbf {x}_{i}$ are required, which reads

17 $$ \frac{\partial\mathbf{f}_{i+1}}{\partial\mathbf{x}_{i}} = \begin{pmatrix} -\mathbf{I} & -h \mathbf{I} & -\frac{1}{2} h^{2}(1-2\beta)\mathbf{I} & \mathbf{0} \\ \mathbf{0} & -\mathbf{I} & -h (1-\gamma) \mathbf{I} & \mathbf{0} \\ \frac{\partial\mathbf{f}_{3,i+1}}{\partial\mathbf{q}_{i}} & \frac {\partial\mathbf{f}_{3,i+1}}{\partial\mathbf{v}_{i}} & \frac{\partial \mathbf{f}_{3,i+1}}{\partial\mathbf{a}_{i}} & \frac{\partial\mathbf {f}_{3,i+1}}{\partial\boldsymbol{\lambda}_{i}}\\ \frac{\partial\mathbf{f}_{4,i+1}}{\partial\mathbf{q}_{i}} & \frac {\partial\mathbf{f}_{4,i+1}}{\partial\mathbf{v}_{i}} & \frac{\partial \mathbf{f}_{4,i+1}}{\partial\mathbf{a}_{i}} & \mathbf{0} \end{pmatrix} $$

with the following partial derivatives of $\mathbf{f}_{3,i+1}$ with respect to $\mathbf{x}_{i}$:

18 $$ \begin{aligned} \frac{\partial\mathbf{f}_{3,i+1}}{\partial\mathbf{q}_{i}} &= \frac{1}{1+\alpha} \mathbf{J_{M}}(t_{i+1}) + \mathbf{J_{C}}(t_{i+1}) - \mathbf{J_{Q}}(t_{i+1}) - \frac{\alpha}{1+\alpha} \bigl[ \mathbf {J_{C}}(t_{i}) - \mathbf{J_{Q}}(t_{i}) \bigr] , \\ \frac{\partial\mathbf{f}_{3,i+1}}{\partial\mathbf{v}_{i}} &= \frac {h}{1+\alpha} \mathbf{J_{M}}(t_{i+1}) + h \mathbf{J_{C}}(t_{i+1}) - h \mathbf{J_{Q}}(t_{i+1}) - \mathbf{J_{V}}(t_{i+1}) + \frac{\alpha}{1+\alpha} \mathbf{J_{V}}(t_{i}), \\ \frac{\partial\mathbf{f}_{3,i+1}}{\partial\mathbf{a}_{i}} &= \frac {h^{2}}{2}(1-2 \beta) \biggl[ \frac{1}{1+\alpha} \mathbf {J_{M}}(t_{i+1}) + \mathbf{J_{C}}(t_{i+1}) - \mathbf {J_{Q}}(t_{i+1}) \biggr] - h(1-\gamma) \mathbf{J_{V}}(t_{i+1}), \\ \frac{\partial\mathbf{f}_{3,i+1}}{\partial\boldsymbol{\lambda}_{i}} &= - \frac{\alpha}{1+\alpha} \mathbf{C}_{\mathbf{q}}^{\mathsf{T}} \bigg\vert _{t=t_{i}}. \end{aligned} $$

The partial derivatives of $\mathbf{f}_{4,i+1}$ with respect to $\mathbf {x}_{i}$ are

19 $$ \begin{aligned} \frac{\partial\mathbf{f}_{4,i+1}}{\partial\mathbf{q}_{i}} &= \frac {1}{\beta h^{2}} \mathbf{C}_{\mathbf{q}} \bigg|_{t=t_{i}}, \\ \frac{\partial\mathbf{f}_{4,i+1}}{\partial\mathbf{v}_{i}} &= \frac {1}{\beta h} \mathbf{C}_{\mathbf{q}} \bigg|_{t=t_{i}}, \\ \frac{\partial\mathbf{f}_{4,i+1}}{\partial\mathbf{a}_{i}} &= \frac {1-2\beta}{2 \beta} \mathbf{C}_{\mathbf{q}} \bigg|_{t=t_{i}}. \end{aligned} $$

In (18) and (19), $h = t_{i+1}-t_{i}$ is the integration step size.

The gradient computation according to (11) requires the following Jacobian matrix:

20 $$ \frac{\partial\mathbf{f}_{i+1}}{\partial\mathbf{u}} = \begin{pmatrix} 0\\ 0\\ \frac{1}{1+\alpha}\frac{\partial(\mathbf{M}\ddot{\mathbf {q}})}{\partial\mathbf{u}} |_{t=t_{i+1}} -\frac{\partial\mathbf{Q}}{\partial\mathbf{u}} |_{t=t_{i+1}} + \frac{\alpha}{1+\alpha}\frac{\partial\mathbf{Q}}{\partial\mathbf {u}} |_{t=t_{i}}\\ 0 \end{pmatrix} . $$

## The discrete adjoints for a simple harmonic oscillator

In this section the discrete adjoint gradient computation is shown on a simple academic example. The reader should get a feel for the proposed method. Let us consider a harmonic one mass oscillator with a linear damping parameter $d$ and a linear stiffness parameter $c$ as a simple example. The goal is to compute the gradient of a cost function of the form (4) with respect to the parameters $c$ and $d$ with the proposed discrete adjoint method. The state vector $\mathbf{x}_{i}=(q_{i},v_{i},a_{i})^{\mathsf{T}}$ consists of the position, velocity, and acceleration of the mass. The systems mass matrix is $\mathbf{M}=m$, the force vector is $\mathbf {Q}_{i}=-c\,q_{i} - d\,v_{i}$, and the system output is $\mathbf {s}(\mathbf{x}_{i})= a_{i}$. For this simple example, the constraint equation (15)_4_ is not required, and therefore the dimension of the Jacobian is reduced. Moreover, all terms related to the constraints equation $\mathbf{C}(\mathbf{q}_{i}) = \mathbf{0}$ are zero. Hence, the Jacobian matrices (16) and (17) can be simply rewritten as

$$ \begin{aligned} \frac{\partial\mathbf{f}_{i}}{\partial\mathbf{x}_{i}} &= \left ( \textstyle\begin{array}{c@{\quad}|@{\quad}c@{\quad}|@{\quad}c} 1 & 0 & -h^{2}\,\beta\\ 0 & 1 & -h \,\gamma\\ 0 & 0 & \frac{m}{1+\alpha} + c\,\beta\,h^{2} + d\,\gamma\,h \end{array}\displaystyle \right ) =: \mathbf{A}, \\ \frac{\partial\mathbf{f}_{i+1}}{\partial\mathbf{x}_{i}} &= \left ( \textstyle\begin{array}{c@{\quad}|@{\quad}c@{\quad}|@{\quad}c} -1 & -h& -\frac{1}{2}h^{2}(1-2\beta) \\ 0 & -1 & -h(1-\gamma) \\ \frac{c}{1+\alpha} & h\,c + \frac{d}{1+\alpha} & \frac{c\, h^{2}}{2}(1-2\beta) + d\,h(1-\gamma) \end{array}\displaystyle \right ) =: \mathbf{B}, \end{aligned} $$

which are constant for linear examples as it is the case here. Inserting both matrices into (9) results in an algebraic system of equations, which must solved successively for $i=N-1,\dots,1$. Starting from $\mathbf{p}_{N}=\mathbf{0}$ and proceeding with

$$ \mathbf{A}^{\mathsf{T}}\mathbf{p}_{i} = -\eta_{i} \begin{pmatrix} 0\\0\\1 \end{pmatrix} \bigl(a_{i} - \bar{a}(t_{i})\bigr) - \mathbf{B}^{\mathsf{T}}\mathbf{p}_{i+1}, $$

the discrete adjoint variables $\mathbf{p}_{i}$ can be computed. Here, $\bar{a}(t_{i})$ denotes the measured accelerations at time $t_{i}$. For simplicity, we set $\alpha=0$ and use an equidistant step size $\eta_{i} = h$, which results in the explicit iteration scheme

$$ \mathbf{p}_{i} = - \begin{pmatrix} 0\\0\\\frac{h}{\tilde{m}}(a_{i} - \bar{a}(t_{i})) \end{pmatrix} - \begin{pmatrix} -1&0&c\\ -h&-1&(d+c\,h)\\ -\frac{h^{2}}{\tilde{m}}&-\frac{h}{\tilde{m}}&\frac{h(d+c\,h)}{\tilde{m}} \end{pmatrix} \,\mathbf{p}_{i+1} $$

with $\tilde{m} = m + \frac{c\,h^{2}}{4} + \frac{d\,h}{2}$. Finally, the gradient can be computed as

$$ \nabla J = \biggl( \frac{\partial J}{\partial\mathbf{u}} \biggr) ^{\mathsf{T}}= \sum _{i=1}^{N-1} \begin{pmatrix} 0 & 0 & q_{i}\\ 0 & 0 & v_{i}\\ \end{pmatrix} \,\mathbf{p}_{i}. $$

The state variables $q_{i}$, $v_{i}$, and $a_{i}$ that are required for the gradient computation must be obtained from a forward simulation of the system in advance. The respective finite difference scheme is not shown here, but can easily derived by specializing (15).

## Example: engine mount

**Fig. 1:**
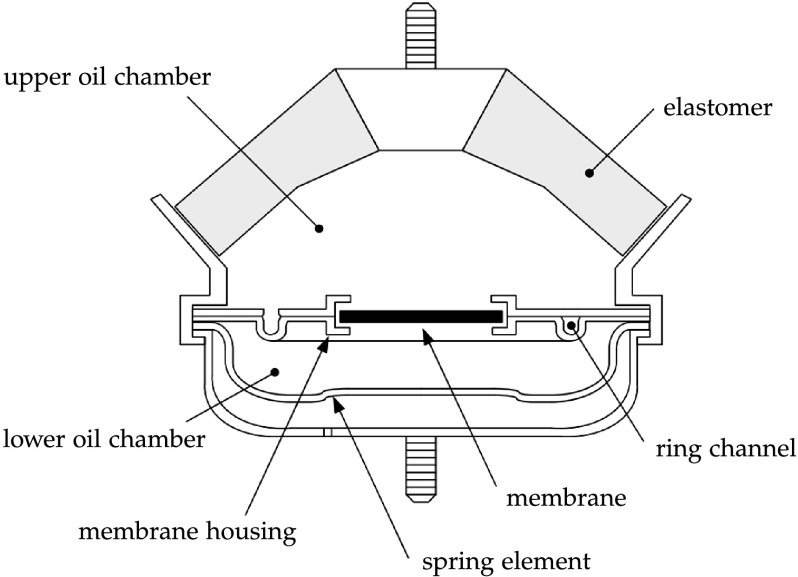
Functional principle of an engine mount as depicted in [2]

As an illustrative example, we consider an engine mount, which is installed in every commercial car. The main purpose of this component is, on the one hand, the damping of low-frequency oscillations and, on the other hand, the isolation of high-frequency vibrations from the chassis produced by the engine. Hence, the frequency selective damping is essential for this component. Further information on hydro-mounts can be found in the work by Amelunxen [2], and its functional principle is shown in Fig. 1.

**Fig. 2:**
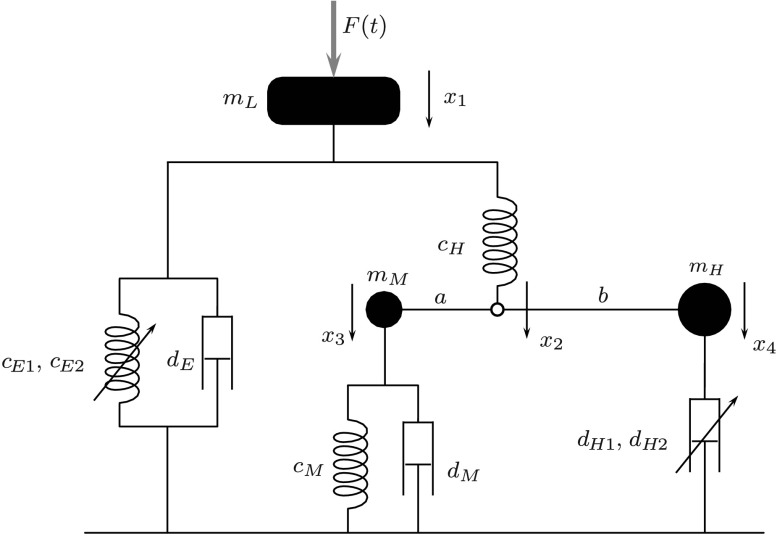
Model of the engine mount [10]

In the development of combustion engines the behavior of the engine mount is of particular interest. Hence, a sufficient accurate mathematical model is important for valid simulation results. In this contribution an analogous model, shown in Fig. 2, is used. Note that in the literature other mathematical formulations can be found (see [2, 9, 17]).

The goal of the present example is to identify a set of parameters such that a measurable output matches a desired trajectory. First of all, we introduce the vector of degrees of freedom $\mathbf{q}=(x_{1}, x_{2}, x_{3}, x_{4})^{\mathsf{T}}$ and a vector of parameters to identify $\mathbf{u}= (c_{E1}, c_{E2}, d_{E}, d_{H2})^{\mathsf{T}}$. In Table 1 the parameters of the model (see Fig. 2) are summarized, and optimal and initial values for the four parameters to identify are given.

**Table 1:** Parameters for numerical simulation

Parameter	Value	
$m_{L}$	20 kg	
$m_{H}$	0.0019 kg	
$m_{M}$	0.002 kg	
$c_{H}$	375 N/mm	
$d_{H1}$	${0.8} \cdot10^{-4}~\mbox{Ns/mm}$	
$c_{M}$	9 N/mm	
$d_{M}$	0.01 Ns/mm	
*a*	95 mm	
*b*	3.6 mm	
*g*	$9.81~\mbox{m/s}^{2}$	
Par. to identify	Optimal value	Initial value
$d_{H2}$	$2 \cdot10^{-9}~\mbox{N}^{3}\,s^{3}/\mbox{mm}^{3}$	${1.2}\cdot10^{-9}~{\mbox{N}^{3}\,\mbox{s}^{3}/\mbox{mm}^{3}}$
$c_{E1}$	$123~\mbox{N/mm}$	$73.8~\mbox{N/mm}$
$c_{E2}$	$2.5~\mbox{N/mm}$	$4~\mbox{N}^{3}/\mbox{mm}^{3}$
$d_{E}$	${5} \cdot10^{-3}~{\mbox{Ns/mm}}$	${5} \cdot 10^{-4}~\mbox{Ns/mm}$

**Fig. 3:**
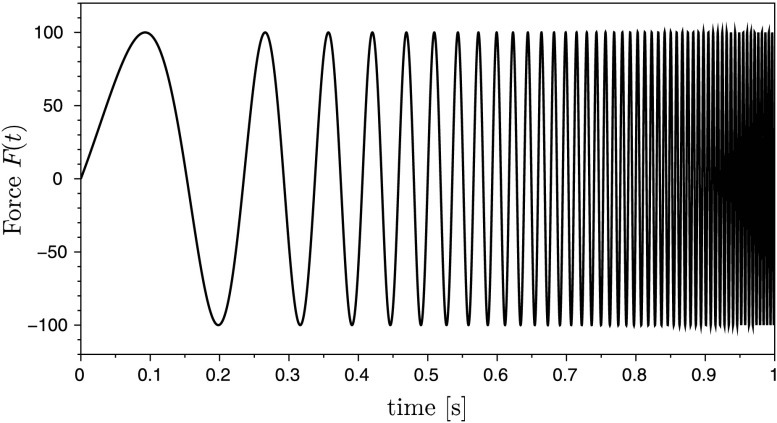
Time-dependent input force $F(t)$

The system equations can be formulated in the form (1). The mass matrix is a constant diagonal matrix given by $\mathbf{M}= \operatorname{diag}(m_{L},0,m_{M},m_{H})$ with a zero mass corresponding to the second degree of freedom $x_{2}$. The external force vector results in

21 $$ \mathbf{Q}= \begin{pmatrix} F(t) + m_{L}\,g - F_{E}(x_{1},\dot{x}_{1}) - c_{H} (x_{1}-x_{2}) \\ c_{H} (x_{1}-x_{2}) \\ - F_{M}(x_{3}, \dot{x}_{3}) \\ - F_{H}(\dot{x}_{4}) \end{pmatrix} $$

with the given time-dependent force

$$ F(t) = A \, \sin \bigl[ \omega_{0} \, C^{t} \,t \bigr] , $$

which is shown in Fig. 3. The function represents an exponential frequency sweep with parameters $\omega_{0}=4\,\pi$ and $C=25$ and the amplitude $A=100~\mbox{N}$. The elastomer force $F_{E}(x_{1},\dot{x}_{1}) = c_{E1} x_{1} + c_{E2} x_{1}^{3} + d_{E} \dot {x}_{1}$ consists of a nonlinear spring and a linear damper, whereas the membrane force $F_{M}(x_{3},\dot{x}_{3}) = c_{M} x_{3} + d_{M} \dot {x}_{3}$ is described by a linear spring and a linear damper. Finally, the hydro-damper force is given by $F_{H}(\dot{x}_{4}) = d_{H1} \dot {x}_{4} + d_{H2} \dot{x}_{4}^{3}$. The algebraic constraint equation

$$ c(\mathbf{q}) = x_{2}\,(a+b) - x_{3}\,b - x_{4} \,a $$

links the redundant generalized coordinates.

**Fig. 4:**
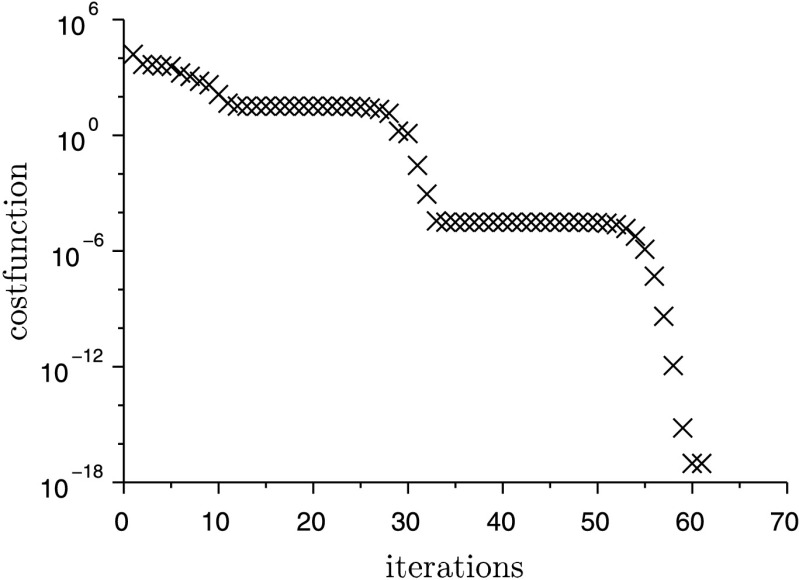
Convergence of the cost function

**Fig. 5:**
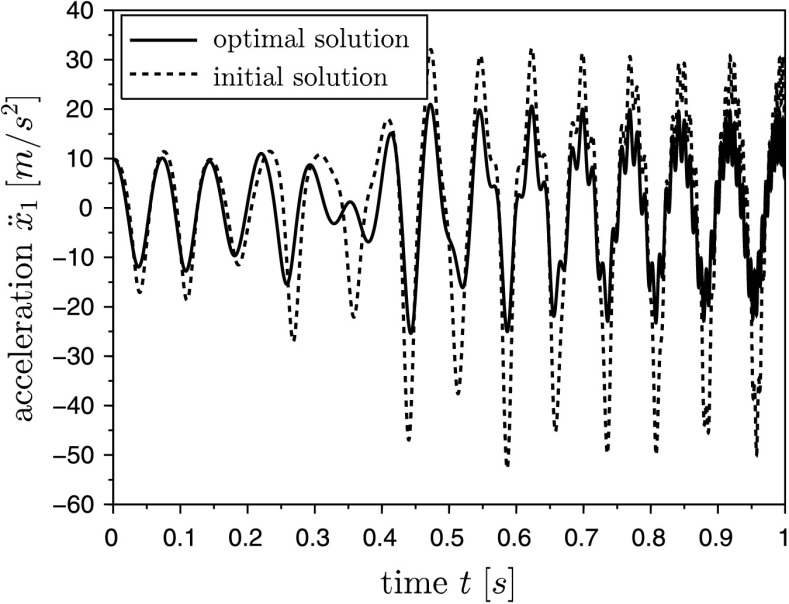
Comparison of the system outputs

**Fig. 6:**
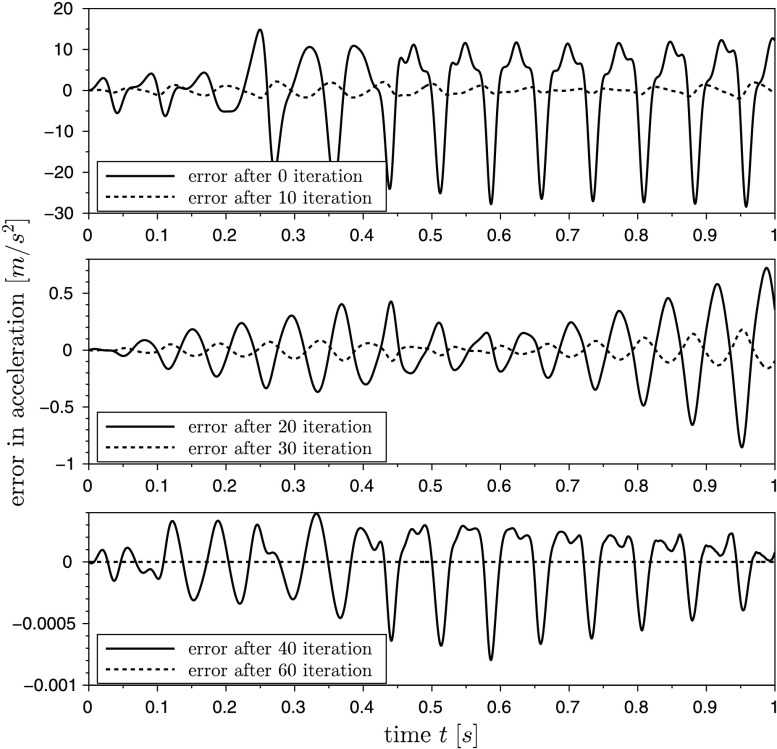
Error plots during parameter identification

The cost function to be minimized is the quadratic deviation of the system output and a measured trajectory $\bar{s}(t)$, which is often called an RMS-error (root-mean-square error). The desired trajectory $\bar{s}(t)$ originates from a simulation of the system equations with the final (optimal) set of parameter $\mathbf{u}_{\text{opt}}$. Instead of using virtual test data, a measured trajectory from a test-bench can be used here. In the present example the system output $s(\ddot{\mathbf{q}})$ is the acceleration $\ddot{x}_{1}$. On the one hand, acceleration sensors are cheaper than other measure equipments, and, on the other hand, the discrete adjoint method in combination with the HHT-solver makes a straightforward implementation possible. In Fig. 5 the comparison between the desired trajectory $\bar{s}(t)$ and the system output $s(\ddot{\mathbf{q}})$ is plotted over the time, in which $s(\ddot{\mathbf{q}})$ results from a simulation with the initial parameter. The large deviation between the initial and the optimal solution results from the incorrect values of the parameters to identify. The reduction of the cost function during the optimization is shown in Fig. 4. After approximately 60 iterations, the value of the cost function is about $10^{-18}$, which means that the deviation of the system output and the desired trajectory is almost zero. In Fig. 6 the RMS-error $e(t) = s(\ddot{\mathbf{q}})-\bar{s}(t)$ is shown during the optimization. It can be seen that the error is reduced significantly with increasing number of iterations.

**Fig. 7:**
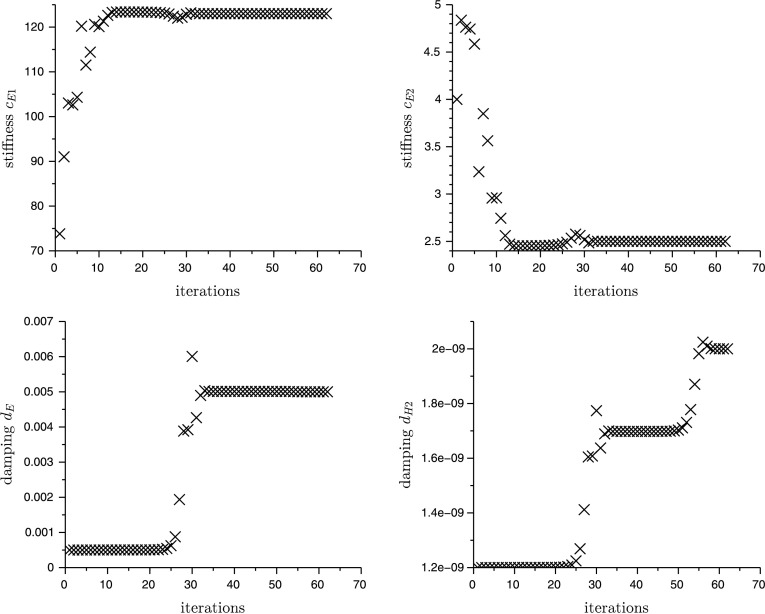
Convergence of the parameter to identify

The convergence of the four parameters over the iterations is shown in Fig. 7. It can be seen that the linear stiffness parameter $c_{E1}$ converges very fast, whereas the cubic damping parameter $d_{H2}$ converges slower.

## Conclusion

In this paper, we show a new approach for the computation of the gradient of a cost function associated with a dynamical system for a parameter identification problem. We present the discrete adjoint method for an implicit discretization scheme and the required Jacobian matrices in detail for the HHT-solver as a representative of a widely used implicit solver in multibody dynamics. Note that the discrete adjoint system depends on the integration scheme of the system equations.

The presented method has two main advantages in comparison with the traditional adjoint method in the continuous case (see, e.g., [11, 20]): First, no separate solver is required to solve the adjoint differential algebraic system backward in time. The computation of the adjoint variables depends only on the recursive iteration scheme used to solve the system equations. Hence, only a system of algebraic equations has to be solved successively. The second advantage is that in combination with the HHT-solver, the cost function may also depend on the accelerations, if the discrete adjoint method is used. The reason is that the accelerations are included in the state vector of the solver method. Hence, the Jacobian matrices that are necessary for the discrete adjoint computations remain similar to the Jacobian matrices that are required for the HHT-solver. Otherwise, in the continuous case, the accelerations are not included in the state vector, but have to be expressed by the motion equations in the cost function, which lead to complex Jacobian matrices [12]. The straightforward and efficient considerations of the acceleration in the cost function have the advantage that the measured signals from acceleration sensors can be used directly for parameter identification in practice. Due to the simple use and low price of acceleration sensors, this strategy is a promising approach in the field of parameter identification.

The theory described in this paper is a powerful tool for parameter identification in time domain. In most cases the results lead to a best-fit solution, which means that high-frequency components with low amplitudes are not considered. However, the discrete adjoint method can also be used to identify parameters influencing the system at special frequencies. Hence, the basic idea is to compute the Fourier coefficients for the relevant oscillations and include the amplitude spectrum in the cost function. In [8] the parameters of a torsional vibration damper of a four-cylinder combustion engine are identified in combination with adjoint Fourier coefficient and the discrete adjoint method.

## References

[1] Alexe M., Sandu A. (2009). On the discrete adjoints of adaptive time stepping algorithms. J. Comput. Appl. Math..

[2] Amelunxen, H.: Fahrdynamikmodelle für Echtzeitsimulationen im komfortrelevanten Frequenzbereich. Ph.D. thesis, Universität Paderborn, Paderborn (2013)

[3] Bryson A.E., Ho Y.C. (1975). Applied Optimal Control.

[4] Eberhard P. (1996). Adjoint variable method for sensitivity analysis of multibody systems interpreted as a continuous, hybrid form of automatic differentiation. Proc. of the 2nd Int. Workshop on Computational Differentiation.

[5] Gavrea B., Negrut D., Potra F.A. (2005). The Newmark integration method for simulation of multibody systems: analytical considerations. Design Engineering, Parts A and B.

[6] Hilber H., Hughes T., Taylor R. (1977). Improved numerical dissipation for time integration algorithms in structural dynamics. Earthq. Eng. Struct. Dyn..

[7] Lauß T., Oberpeilsteiner S., Steiner W., Nachbagauer K. (2016). The discrete adjoint gradient computation for optimization problems in multibody dynamics. J. Comput. Nonlinear Dyn..

[8] Lauß T., Oberpeilsteiner S., Steiner W., Nachbagauer K., Reichl S. (2017). Parameter identification of a torsional vibration damper in frequency domain using adjoint Fourier coefficients. Proceedings of the ECCOMAS Thematic Conference Multibody Dynamics 2017.

[9] Mitschke M., Wallentowitz H. (2014). Dynamik der Kraftfahrzeuge.

[10] Mrazek, T.: Modellierung nichtlinearer Elemente zur Schwingungsdämpfung in Mehrkörpersystmen. Ph.D. thesis, Johannes Kepler Universität, Linz (2005)

[11] Nachbagauer K., Oberpeilsteiner S., Sherif K., Steiner W. (2015). The use of the adjoint method for solving typical optimization problems in multibody dynamics. J. Comput. Nonlinear Dyn..

[12] Nachbagauer K., Oberpeilsteiner S., Steiner S. (2015). Enhancement of the adjoint method by error control of accelerations for parameter identification in multibody dynamics. Univ. J. Control Autom..

[13] Negrut D., Rampalli R., Ottarsson G., Sajdak A. (2007). On an implementation of the Hilber–Hughes–Taylor method in the context of index 3 differential-algebraic equations of multibody dynamics (DETC2005-85096). J. Comput. Nonlinear Dyn..

[14] Newmark N. (1959). A method of computation for structural dynamics. J. Eng. Mech. Div..

[15] Oberpeilsteiner, S., Lauß, T., Nachbagauer, K., Steiner, W.: Optimal input design for multibody systems by using an extended adjoint approach. Multibody Syst. Dyn., 1–12 (2016) 10.1007/s11044-016-9541-8 PMC5393295 28473738

[16] Petzold L., Li S., Cao Y., Serban R. (2006). Sensitivity analysis for differential-algebraic equations and partial differential equations. Comput. Chem. Eng..

[17] Pfeffer P., Hofer K. (2002). Einfaches nichtlineares Modell für Elastomer und Hydrolager zur Optimierung der Gesamtfahrzeug-Simulation. ATZ, Automobiltech. Z..

[18] Schaffer, A.: On the adjoint formulation of design sensitivity analysis of multibody dynamics. Ph.D. thesis, University of Iowa (2005)

[19] Serban R., Freeman J. (2001). Identification and identifiability of unknown parameters in multibody dynamic systems. Multibody Syst. Dyn..

[20] Steiner W., Reichl S. (2012). The optimal control approach to dynamical inverse problems. J. Dyn. Syst. Meas. Control.

[21] Vyasarayani C., Uchida T., McPhee J. (2012). Nonlinear parameter identification in multibody systems using homotopy continuation. J. Comput. Nonlinear Dyn..

